# Large‐scale generation of megakaryocytes from human embryonic stem cells using transgene‐free and stepwise defined suspension culture conditions

**DOI:** 10.1111/cpr.13002

**Published:** 2021-02-21

**Authors:** Bowen Zhang, Xumin Wu, Guicheng Zi, Lijuan He, Sihan Wang, Lin Chen, Zeng Fan, Xue Nan, Jiafei Xi, Wen Yue, Lei Wang, Liu Wang, Jie Hao, Xuetao Pei, Yanhua Li

**Affiliations:** ^1^ Experimental Hematology and Biochemistry Lab Beijing Institute of Radiation Medicine Beijing China; ^2^ South China Research Center for Stem Cell & Regenerative Medicine SCIB Guangzhou China; ^3^ Stem Cell and Regenerative Medicine Lab Institute of Health Service and Transfusion Medicine Beijing China; ^4^ State Key Laboratory of Stem Cell and Reproductive Biology Institute of Zoology Chinese Academy of Sciences Beijing China; ^5^ Institute for Stem Cell and Regeneration Chinese Academy of Sciences Beijing China; ^6^ National Stem Cell Resource Center Chinese Academy of Sciences Beijing China; ^7^ University of Chinese Academy of Science Beijing China

**Keywords:** differentiation, human embryonic stem cells, megakaryocytes, suspension culture

## Abstract

**Objectives:**

Ex vivo engineered production of megakaryocytes (MKs) and platelets (PLTs) from human pluripotent stem cells is an alternative approach to solve shortage of donor‐donated PLTs in clinics and to provide induced PLTs for transfusion. However, low production yields are observed and the generation of clinically applicable MKs and PLTs from human pluripotent stem cells without genetic modifications still needs to be improved.

**Materials and Methods:**

We defined an optimal, stepwise and completely xeno‐free culture protocol for the generation of MKs from human embryonic stem cells (hESCs). To generate MKs from hESCs on a large scale, we improved the monolayer induction manner to define three‐dimensional (3D) and sphere‐like differentiation systems for MKs by using a special polystyrene CellSTACK culture chamber.

**Results:**

The 3D manufacturing system could efficiently generate large numbers of MKs from hESCs within 16‐18 days of continuous culturing. Each CellSTACK culture chamber could collect on an average 3.4 × 10^8^ CD41^+^ MKs after a three‐stage orderly induction process. MKs obtained from hESCs via 3D induction showed significant secretion of IL‐8, thrombospondin‐1 and MMP9. The induced cells derived from hESCs in our culture system were shown to have the characteristics of MKs as well as the function to form proPLTs and release PLTs. Furthermore, we generated clinically applicable MKs from clinical‐grade hESC lines and confirmed the biosafety of these cells.

**Conclusions:**

We developed a simple, stepwise, 3D and completely xeno‐free/feeder‐free/transgene‐free induction system for the generation of MKs from hESCs. hESC‐derived MKs were shown to have typical MK characteristics and PLT formation ability. This study further enhances the clinical applications of MKs or PLTs derived from pluripotent stem cells.

## Graphical abstract

We developed a simple, stepwise, three‐dimensional (3D) and completely xeno‐free/feeder‐free/transgene‐free induction system for generation of MKs from human embryonic stem cells (hESCs). hESC‐derived MKs were shown to have the typical MK characteristics and PLT formation ability. This study further enhances the clinical application of MKs or PLTs derived from pluripotent stem cells.

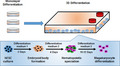

## 1 INTRODUCTION

Platelet (PLT) transfusion is an important therapeutic approach for patients with life‐threatening thrombocytopenia. However, there is a severe supply‐demand imbalance for PLT transfusion due to the shortage of donations from human volunteers and the increasing demand for the transfusion process in clinics.^1^, ^2^ PLTs are primarily generated from megakaryocytes (MKs) and their progenitors in the bone marrow (BM) after birth.^3^, ^4^, ^5^ It is difficult, rather impossible, to obtain MKs from normal BM for in vitro PLT production due to their rarity and the painful surgery manipulation. To solve the PLT shortage problem, an alternative approach involving ex vivo engineered production of MKs from human stem cells can be applied. Along with the subsequent generation of PLTs from MKs for transfusion therapy, the induced MKs or their progenitors directly infused into human patients have been accepted as a therapeutic strategy for prophylaxis or treatment of thrombocytopenia.^6^, ^7^, ^8^ Thus, stem cell‐derived MKs and PLTs serve as advanced therapeutic and medicinal products.

The generation of functional MKs and PLTs from hematopoietic stem and progenitor cells (HSPCs) derived from cord blood and BM has been successfully employed in laboratories.^9^, ^10^ The limited number and proliferation capacity of HSPCs in these samples have impeded the development of large‐scale and standard production of MKs and PLTs. It is known that human pluripotent stem cells, including human embryonic stem cells (hESCs) and induced pluripotent stem cells, have an indefinite expansion capacity. These cells might act as ideal seed cells for in vitro production of large‐scale and donorless MKs and PLTs.^11^, ^12^ Several studies have shown that hESCs can produce MKs and PLTs using stromal cell co‐cultures or embryonic body induction process.^13^, ^14^, ^15^ Recently, expandable MK cell lines by overexpression of several genes in pluripotent stem cells have been accepted as the most promising cellular resource for clinically applicable and large‐scale generation of PLTs.^16^, ^17^ However, low yields of such clinically applicable MKs and PLTs are obtained from hESCs without undergoing genetic modification and the process needs to be further improved.

Here, we defined an optimal, stepwise, and completely defined xeno‐free culture method for the generation of MKs from hESCs. For large‐scale generation of MKs from hESCs, we improved the monolayer induction process to define three‐dimensional (3D) and sphere‐like differentiation models for MKs during the whole process by using a polystyrene CellSTACK culture chamber. The induced cells derived from hESCs in our culture system were shown to exhibit the characteristics of MKs, as well as, proPLT formation and PLT release. The 3D induction protocol could generate (3.4 ± 2.5) × 10^8^ CD41a^+^ MKs from hESCs per CellSTACK culture chamber within 16‐18 days of cell culturing. This study could aid in the automatic production of MKs using this cell culture chamber and could also assist in the clinical application of hESC‐derived MK infusion.

## 2 MATERIALS AND METHODS

### 2.1 Human embryonic stem cell culture

The H9 hESC line (from WiCell Research Institute) was cultured in a chemically defined mTeSR1^TM^ medium on matrigel‐coated wells as previously described.^18^ Medium changes were performed daily, and confluent cultures were passaged every 4 ‐ 6 days using ReLeSR^TM^.

The clinical hESC line (Q‐CTS‐hESC‐2) was prepared as described previously.^19^ Clinical hESCs were cultured in xeno‐free Essential 8^TM^ (E8) medium on vitronectin (VTN‐N)‐coated plates (1 µg/cm^2^). Cells were passaged every 4‐6 days using Versene.

All cultures were maintained at 37°C in a 5% CO_2_ incubator (Thermo Fisher Scientific). Mycoplasma contamination was tested every 2 weeks using the MycoAlert^TM^ Mycoplasma Detection Kit (Lonza).

### 2.2 Megakaryocyte differentiation

For monolayer induction, single‐cell suspensions of hESCs were plated at a density of 1 × 10^4^ cells/cm^2^ onto 6‐well plates. After 24 hours (day 0), the medium was changed to differentiation medium 1 consisting of Advanced Dulbecco's medium/F12 supplemented with 1 × GlutaMAX^TM^, 50 μg/mL L‐ascorbic acid 2‐phosphate (AA2P), BMP4 (25 ng/mL), bFGF (25 ng/mL), Activin A (25 ng/mL) and CHIR99021 (2 μmol/L). On day 2, the medium was changed to differentiation medium 2 consisting of Advanced Dulbecco's medium/F12 supplemented with 1 × GlutaMAX^TM^, 50 μg/mL AA2P, VEGF (50 ng/mL), bFGF (25 ng/mL) and TGFβ inhibitor SB431542 (5 μmol/L). On day 6, the induced cells were dissociated with 1 × TrypLE Select. Purified CD34^+^ cells were enriched using an MACS separation column and replated at a density of 5 × 10^4^ cells/cm^2^ onto 12‐well plates in differentiation medium 3 consisting of BEL medium supplemented with SCF (50 ng/mL), TPO (40 ng/mL), IL‐3 (20 ng/mL), Flt3L (20 ng/mL), VEGF (20 ng/mL), IGF1 (20 ng/mL), bFGF (10 ng/mL), IL‐11 (10 ng/mL), and SB431542 (10 μmol/L). The medium was changed daily during the induction process. The BEL medium was prepared according to the references.^20^, ^21^ The composition of the BEL medium is listed in Table S1. Main reagents used for hESC culture and differentiation are described in detail in Table S2.

For induction of the 3D suspension culture, hESC colonies were dissociated into single cells by Accutase^TM^ on day −1. After harvesting the cells and counting them, the cells were resuspended in culture medium supplemented with the Rho kinase (ROCK) inhibitor Y27632 (10 μmol/L) and plated at a density of 10^5^ cells/mL in an ultra‐low CellSTACK^TM^ culture chamber. On day 0, hESC aggregates were collected and resuspended in differentiation medium 1. On day 2, the EBs were collected and resuspended in differentiation medium 2. On day 6, the EBs were collected and resuspended in differentiation medium 3. The medium was changed daily during the induction process. On day 16‐18, the suspended single cells generated from adherent cells or EBs were collected by passing through a 40 μm cell strainer for hematopoietic cell analysis.

### 2.3 Flow cytometry analysis

For surface marker analysis, cultured cells or PLTs in the culture supernatant were collected by centrifugation and labelled with fluorescein‐conjugated antibodies in PBS for 30 minutes at room temperature in dark and were then analysed using a flow cytometer (BD Aria).

For α‐granule release analysis, human blood PLTs or hESC‐PLTs were treated with thrombin (2 U/mL) for 20 minutes at 37°C before labelling with CD41a, CD42b and CD62P antibodies in modified Tyrode's buffer. The samples were then analysed using a flow cytometer.

For polyploidy analysis, the cultured cells were collected and resuspended in PBS containing Hoechst 33342 (5 μg/mL) and then labelled with antibodies against CD41a and CD42b for 60 minutes at 37°C in dark. The cells were pelleted and resuspended in PBS and analysed using a flow cytometer.

Flow cytometry data were analysed using the FlowJo software. The information of antibodies is described in detail in Table S2.

### 2.4 Formation of platelet microaggregates

Human blood PLTs and hESC‐derived PLTs were resuspended in modified Tyrode's buffer and labelled with a PKH26 Fluorescent Cell Linker (10 µmol/L, Sigma‐Aldrich). Human blood PLTs (labelled with PKH67, 6 × 10^7^) were mixed with fluorescence‐labelled human blood PLTs (3 × 10^6^) or hESC‐PLTs (3 × 10^6^), treated with thrombin (5 U/mL), and stirred at 1200 rpm at 37°C to trigger PLT aggregation. PLT microaggregates in 50 µL buffer were spread onto glass slides and visualized under a fluorescence microscope (GE Healthcare).

### 2.5 CFU‐MK assay

Colony‐forming unit (CFU)‐MK assay was performed by plating 2 × 10^4^ cells/well from differentiated MKs following manufacturer's protocol (STEMCELL). Cells were incubated at 37°C in a 5% CO_2_ incubator, and MK colonies were fixed and stained with CD41 antibody at day 14. CD41^+^ colonies were counted and classified following manufacturer's criteria.

### 2.6 Statistical analysis

Data are presented as mean ± SEM. Statistical analyses were performed using the GraphPad Prism software. The statistical significance of the differences was determined using unpaired two‐tailed Student's *t*‐tests. Values with *P* < .05 were considered statistically significant.

## 3 RESULTS

### 3.1 Generation of high purity megakaryocytes in an optimized stepwise 2D induction process

**FIGURE 1:**
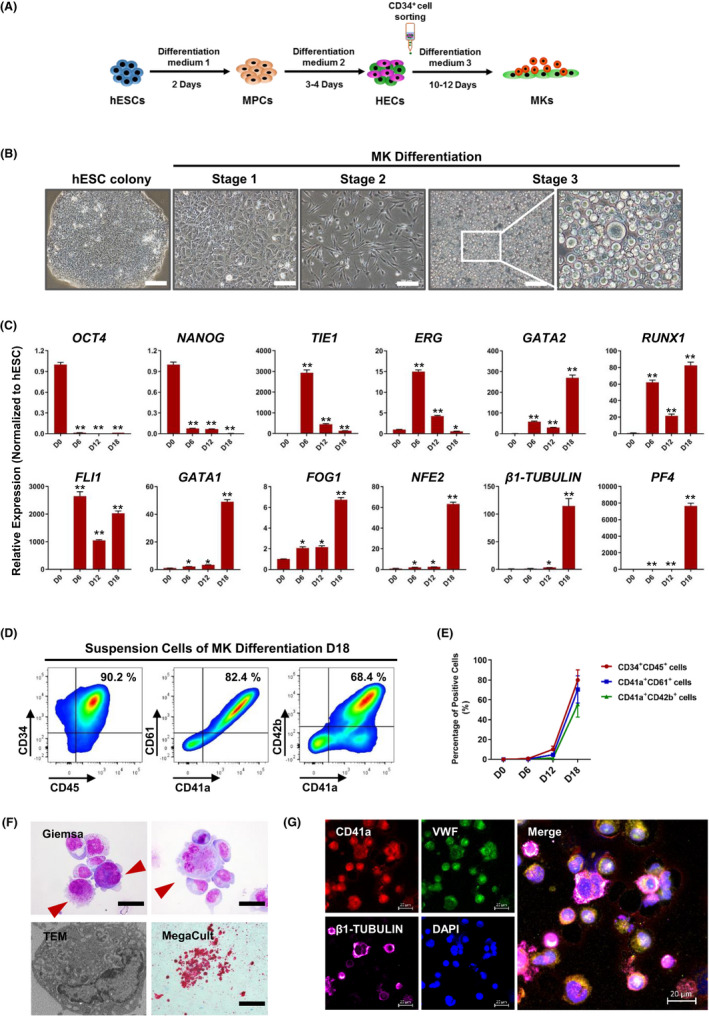
Generation of megakaryocytes (MKs) derived from human embryonic stem cells. A, A schematic showing megakaryocytic differentiation of hESCs through a monolayer induction method. B, Phase contrast images of hESC cultures at days 0 (hESC colony), 2 (Stage 1), 6 (Stage 2) and 18 (Stage 3) of megakaryocytic differentiation. Scale bars represent 100 μm. C, Quantitative RT‐PCR analysis of genes associated with pluripotency and megakaryocytic development expressed in hESC cultures undergoing megakaryocytic differentiation. Relative expression levels are normalized to hESC levels. *P < .05, **P < .01 compared with hESCs. D, Flow cytometric analysis of expression of hematopoietic markers (CD34 and CD45) and MK markers (CD41a, CD61 and CD42b) in suspension cells on day 18 of differentiation. E, The kinetics of the appearance of hematopoietic progenitor (CD34^+^CD45^+^) and MK (CD41a^+^CD61^+^ and CD41a^+^CD42b^+^) markers during differentiation experiments (n = 4 independent experiments). F, Characterization of MKs from hESCs. Giemsa: Scale bars represent 25 μm. TEM: Direct Mag = 10 000 ×. MegaCult: Scale bars represent 200 μm. G, Immunofluorescence of CD41a, VWF and β1‐TUBULIN in hESC‐derived MK cells. Scale bars represent 20 μm

To efficiently generate MKs from hESCs in a xeno‐free system, we developed a monolayer, adherent and stepwise induction protocol without feeder cell corporation (Figure 1A). The hESCs (Q‐CTS‐hESC‐2 and H9 cells) were digested into single cells and plated on wells at a low seeding density (1 × 10^4^ cells/cm^2^). These hESCs presented typical well‐defined colony morphology, normal karyotype, positive alkaline phosphatase staining, and expressed the pluripotency‐related proteins OCT4, SOX2, and NANOG (Figure 1B and Figure S1A‐C). After induction for 2 days in differentiation medium 1, the cells exhibited high expression levels of key mesoderm marker proteins, such as BRACHYURY (BRA) and ROR2 (Figure S1D). To refine the hemogenic differentiation potential of these mesoderm progenitor cells, the cells were subsequently cultured using optimized cytokines and small molecule supplements (differentiation medium 2 supplemented with bFGF, VEGF and SB431542) for 3‐4 days. After this induction period, the differentiated cells were digested and sorted for CD34^+^ cells to enrich for hemogenic cells. The isolated CD34^+^ cells were subsequently cultured in differentiation medium 3 to attain hematopoietic and MK specification. After 10‐12 days of induction, more suspension cell clusters were grown from the adherent cells (Figure 1B). We detected dynamic gene expression patterns in pluripotent, hematopoietic and MK markers during the entire differentiation process. The expression of pluripotent genes, *OCT4* and *NANOG*, was rapidly and significantly decreased during MK induction. In contrast, key transcription factors and marker genes for hematopoietic cells and MKs, such as *GATA2, RUNX1, FLI1, GATA1*, *FOG1*, *NFE2*, *β1‐TUBULIN* and *PF4*, reached higher expression levels after stage 3 of the induction process (Figure 1C). We then analysed the percentage of hematopoietic cells, MK progenitor cells and MK cells after the specification process using flow cytometry. The results showed that the monolayer and stepwise induction method could generate high purity of hematopoietic progenitor cells and MK cells within 18 days after induction, with (80.0 ± 10.1) % CD34^+^CD45^+^ cells, (70.5 ± 13.8) % CD41a^+^CD61^+^ cells and (55.5 ± 13.0) % CD41a^+^CD42b^+^ cells on day 18 of 3‐stage differentiation, respectively (Figure 1D,E). In these suspension cells, we detected the presence of large cells with polylobulated nuclei structures. Electron microscopic observations of induced cells demonstrated typical MK organelles. Functionally, by colony‐forming unit (CFU)‐MK assay, we observed the emergence of colonies with a high expression of CD41 on day 14 (Figure 1F). Immunofluorescence staining results indicated that most of the differentiated cells co‐expressed CD41a and VWF, approximately 55 % MK‐like cells with a high expression of β1‐TUBULIN, a critical feature of MKs capable of PLT production function (Figure 1G). However, the monolayer differentiation protocol led to a low MK yield at approximately 5 MKs/hESCs.

### 3.2 Large‐scale generation of megakaryocytes by a 3D suspension induction method

**FIGURE 2:**
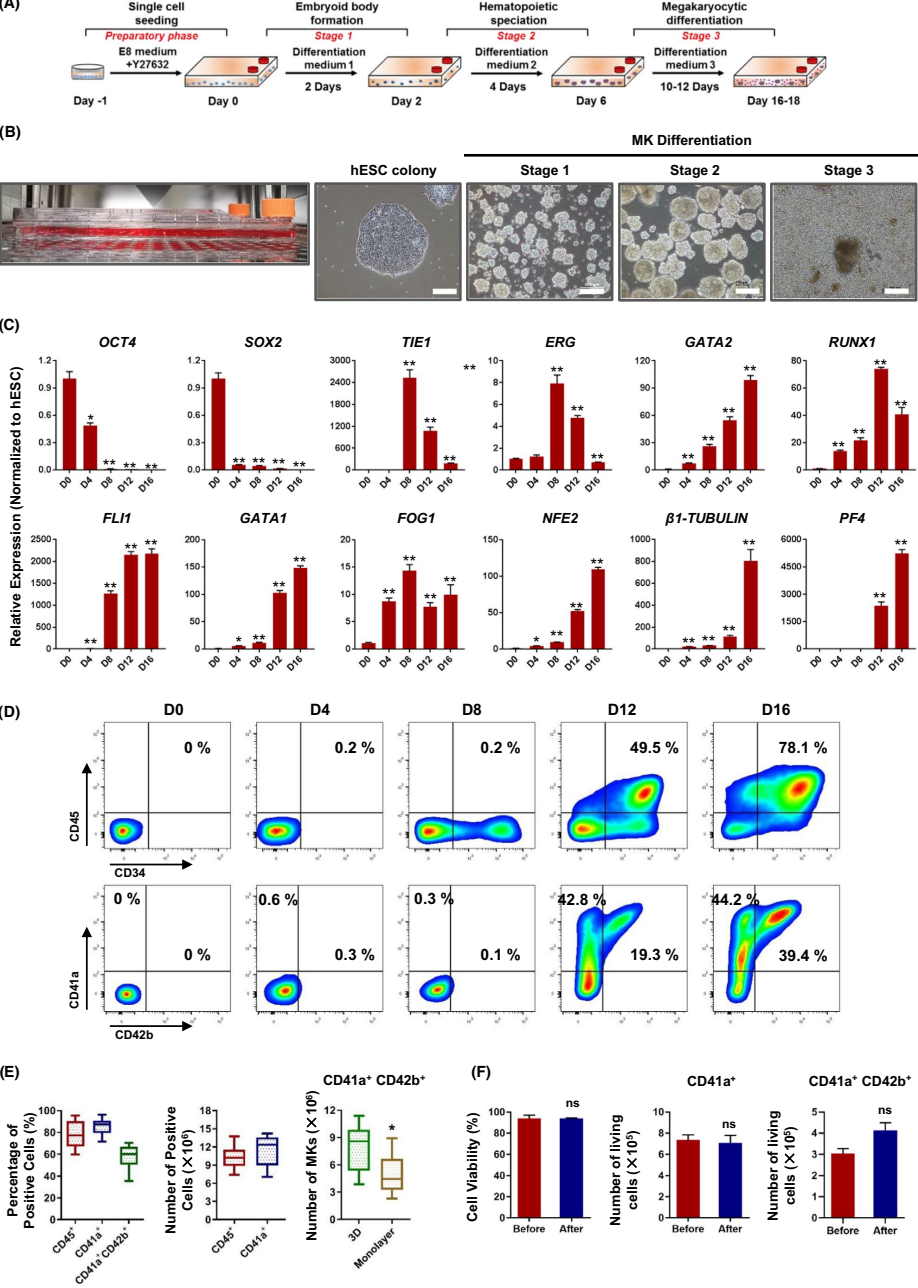
Large‐scale generation of MKs through 3D suspension induction. A, A schema of hESC megakaryocytic differentiation method based on EB formation. B, Phase contrast images of hESC‐derived EB cultures grown at the indicated stages of megakaryocytic differentiation. Scale bars represent 200 μm. C, Quantitative RT‐PCR analysis of pluripotency and megakaryocytic marker genes expressed in EB cultures at the indicated differentiation stages. Relative expression is normalized to hESCs. *P < .05, **P < .01 compared with hESCs. D, The kinetics of hematopoietic and megakaryocyte differentiation from hESCs at the indicated days. E, Analysis of percentages and changes in absolute cell numbers among CD45^+^, CD41a^+^, and CD41a^+^CD42b^+^ cells (from 3D and monolayer induction) generated on day 16. Data are shown for 12 independent experiments. F, Analysis of cell survival rates and megakaryocyte marker expression levels after cryopreservation and resuscitation cycles

The manipulation process for two‐dimensional (2D) MK generation was complex and produced a low yield. To obtain large numbers of MKs for clinical application, we further developed a 3D suspension differentiation protocol using a CellSTACK culture chamber. The CellSTACK culture chamber has a large culture bottom area of 636 cm^2^ and an ultra‐low attachment surface, which is recommended for the large‐scale expansion of suspension cells (Figure 2A). To initiate a suspension culture process, the hESC colonies were digested into single cells and transferred onto a polystyrene CellSTACK chamber supplemented with E8 medium and Y27632 for 1 day (Figure 2A). Single hESC began to aggregate and form embryoid bodys (EBs) in the suspension state (Figure 2A). These EBs were subsequently induced to differentiate into mesoderm progenitor cells, hemogenic endothelium cells, hematopoietic progenitor cells, and MKs in a stepwise induction medium in the suspended 3D state without a CD34 immunobead selection step. During the third stage of differentiation, the EBs gradually produced large numbers of round single cells around them (Figure 2B). The expression of the pluripotent genes, *OCT4* and *SOX2*, was rapidly and significantly decreased during the second stage of induction process and was undetectable in the suspended single cells produced by EBs on day 16 (Figure 2C). Transcription factors associated with hemogenic endothelium and early hematopoiesis development, *TIE1* and *ERG* expression increased to peak during stage 2 differentiation. The gene expression levels of the key hematopoietic and MK transcriptional factors, such as *GATA2*, *RUNX1*, *FLI1*, *GATA1*, *FOG1* and *NFE2*, were significantly upregulated compared to day 0 (Figure 2C). MK specific marker genes, *β1‐TUBULIN* and *PF4*, were notably increased on day 12 and reached the highest expression level after three stages of induction (Figure 2C). Hematopoietic and MK cell surface markers could not be detected during the first and second stages of induction. During the third stage of induction, the CD34^+^CD45^+^ hematopoietic cell percentage and numbers showed significant increases and CD45^+^ cell percentages reached (79.5 ± 12.9) % at day 16 (Figure 2D,E and Figure S2). Notably, among the suspended single cells generated from the EBs, (85.2 ± 7.2) % of the cells expressed CD41a and (53.6 ± 17.5) % of the cells co‐expressed the mature MK markers CD41a and CD42b (Figure 2D,E). The 3D differentiation protocol led to a cellular yield of (11.4 ± 2.5) × 10^6^ CD41a^+^ MKs/1 × 10^6^ hESCs and (7.3 ± 3.0) × 10^6^ CD41a^+^ CD42b^+^ MKs/1 × 10^6^ hESCs,0.6‐fold higher than the CD41a^+^CD42b^+^ cellular yield of monolayer induction (Figure 2E), which also presented high cellular viability and maintained the cell numbers both before and after preservation for 6 months in liquid nitrogen (Figure 2F). Within 16‐18 days after stepwise induction, (3.4 ± 2.5) × 10^8^ CD41a^+^ MKs could be obtained from each CellSTACK chamber. Approximately 1 × 10^9^ MKs could be generated using 4 CellSTACK chambers. The induction procedure was easy to manipulate and relatively simple compared to the monolayer adherent induction method. More importantly, large numbers of human‐induced MKs could be produced within a 16‐18‐day culture period from hESCs by using more CellSTACK chambers.

### 3.3 Characteristics of MKs derived from hESCs after 3D induction

**FIGURE 3:**
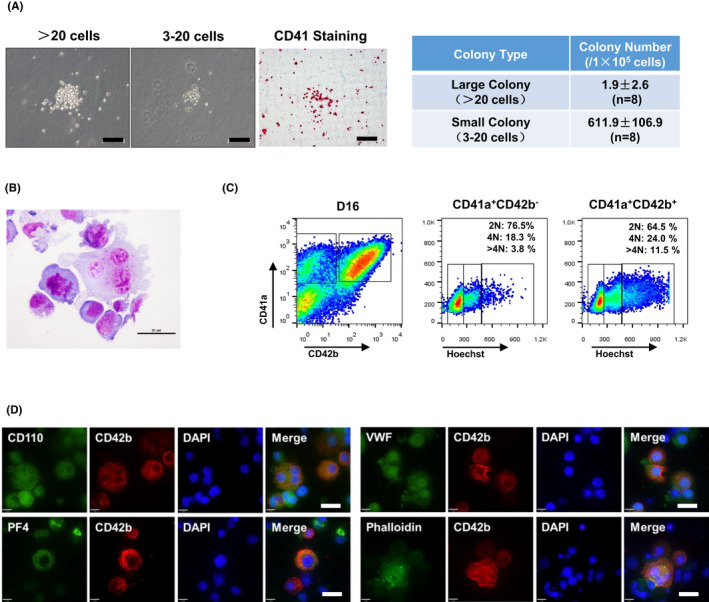
Characterization of human embryonic stem cell‐derived MKs. A, Representative images of CFU‐MK colonies on day 16 of MK differentiation. Scale bars represent 20 μm. B, Giemsa staining of the suspended single cells from day 16 of MK differentiation. Scale bars represent 25 μm. C, DNA ploidy analysis by flow cytometry analysis of MK cells at day 16. D, Immunofluorescence of CD110, CD42b, PF4, VWF proteins, and phalloidin staining in hESC‐derived MK cells. Scale bars represent 20 μm

We evaluated the characteristics of MKs derived from hESCs after the 3D induction process. The CFU‐MK assay showed that these cells could generate approximately (613.8 ± 109.0) MK colonies/1 × 10^5^ suspended single cells, which mainly consisted of small colonies (Figure 3A). Wright‐Giemsa staining of the suspended single cells generated from EBs on day 16 revealed a typical mixture of megakaryoblasts with large peripheral nuclei and mature MKs with larger and polyploid morphology, granular accumulation in the cytoplasm, and small protrusion on the membrane (Figure 3B). We also evaluated the polyploidy of the differentiated cells on day 16. The flow cytometric analysis results showed that approximately 64.5 % of the CD41a^+^CD42b^+^ cells were 2N, 24 % of the CD41a^+^CD42b^+^ cells were 4N, and 12 % of the cells were >4N (Figure 3C). The percentage of >4N cells in CD41a^+^CD42b^‐^ cells was less than those in the CD41a^+^CD42b^+^ cells, suggesting that a large percentage of immature MKs in nature were generated in the 3D induction system. We performed immunofluorescence staining to detect the characteristic MK protein expression in these cells. We found that the differentiated CD42b^+^ MKs expressed the TPO receptor CD110, PF4, VWF and phalloidin at high levels (Figure 3D), indicating that some of the induced MKs reached later stages of maturity. These results indicated that the 3D suspension induction protocol generated MKs at different developmental stages with obvious MK characteristics.

### 3.4 Cytokine expression and secretion by MKs derived from hESCs in a 3D‐ induced manner

**FIGURE 4:**
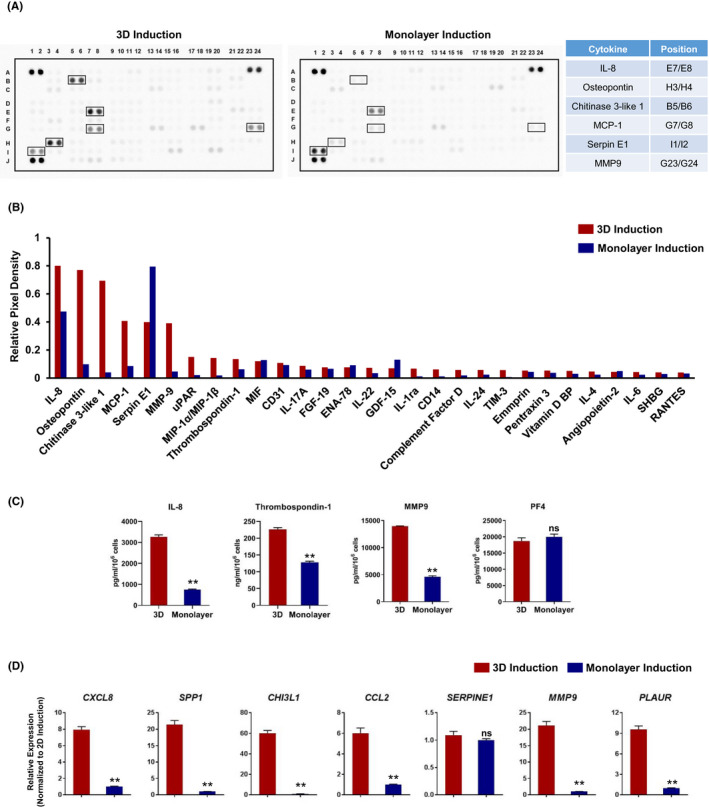
Analysis of cytokine secretion of MK cells from monolayer and 3D methods. A, Images of protein array analysis of MK cell culture supernatants from monolayer or 3D differentiation methods. B, Levels of indicated cytokines in cell culture supernatants of MK cells from monolayer or 3D differentiation methods. C, Secreted cytokine levels in the culture supernatants of MK cells from monolayer or 3D differentiation methods were detected by ELISA. **P < .01 compared with 3D group. D, Quantitative RT‐PCR analysis of genes associated with the indicated cytokine transcription. **P < .01 compared with 3D group

It has been reported that MKs and PLTs can secret various cytokines with multiple functions.^22^, ^23^, ^24^, ^25^, ^26^ To dissect the secretion of cytokines by these differentiated MKs derived from hESCs, we profiled the supernatants from the 3D or monolayer‐induced cells using a cytokine array. We found that over 30 cytokines were secreted by these differentiated MKs (Figure 4A,B). Of note, MKs obtained from hESCs via 3D induction showed increased secretion of IL‐8, osteopontin, Chitinase3‐like 1, MCP‐1, MMP9, uPAR, MIP‐1α/MIP‐1β, Thrombospondin‐1, IL‐22, IL‐1ra, CD14, IL‐24, TIM‐3 and IL‐6 compared to cells from the monolayer induction group (Figure 4A,B). We next employed ELISA to determine the secretion of several key cytokines by these differentiated MKs. We confirmed that the IL‐8 and Thrombospondin‐1 levels were significantly increased in the supernatants from 3D‐induced MKs compared to monolayer‐induced MKs (IL‐8, (3294.6 ± 200.5) pg/mL vs (807.0 ± 56.6) pg/mL; Thrombospondin‐1, (237.9 ± 10.3) ng/mL vs. (134.6 ± 6.9) ng/mL, respectively) (Figure 4C). We observed higher MMP9 secretion in the supernatants from the differentiated cells derived from hESCs after 3D induction than in the monolayer induction group (Figure 4C). Both the MKs from the 3D induction group and monolayer induction group showed high levels of PF4 secretion (Figure 4C). The significant increase in *CXCL8* (encoding IL‐8), *SPP1*(encoding osteopontin), *CHI3L1*, *CCL2* (encoding MCP1), *MMP9* and *PLAUR* (encoding uPAR) gene expression could be detected in the MKs derived from hESCs via 3D induction compared to that in the cells via monolayer induction by using Q‐PCR (Figure 4D). These results provide persuasive evidence that numerous cytokines are synthesized and secreted by MKs derived from hESCs, and the MKs obtained from the 3D induction process were more mature with higher levels of MK‐characteristic cytokine secretion.

### 3.5 Evaluation of clinically‐applicable MKs derived from hESCs

**FIGURE 5:**
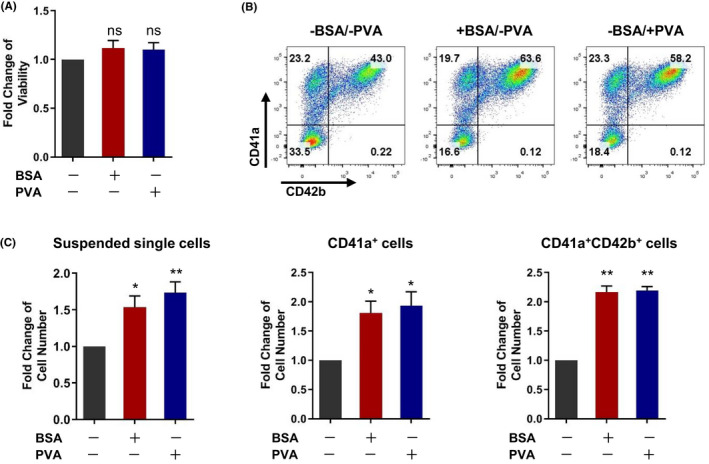
Replacement of bovine serum albumin with polyvinyl alcohol during MK differentiation from hESCs. A, Analysis of relative viability changes of suspended cells on day 18 based on treatment with BSA or PVA during the stage 3 differentiation process. Relative fold changes were normalized to the stage 3 ‐BSA‐PVA differentiation medium. B, Representative FACS profiles of cells harvested on day 18. Cells were treated with or without BSA or PVA during stage 3 differentiation. C, Analysis of changes in relative cell numbers of suspended cells, CD41a^+^ cells, and CD41a^+^CD42b^+^ cells on day 18 based on treatment with BSA or PVA during stage 3 differentiation process. Relative fold changes were normalized to the type of stage 3 ‐BSA/‐PVA differentiation medium. *P < .05, **P < .01 compared with ‐BSA/‐PVA group

To generate clinically applicable MKs from hESCs, the use of animal resources should be avoided in the agents used in the differentiation medium. BSA is a xenogeneic protein and is not biosafe for the preparation of clinical‐grade cell products. To establish a xeno‐free induction medium, we used polyvinyl alcohol (PVA) to replace BSA, a critical component in differentiation medium 3, to nourish the differentiated cells. We found that both BSA and PVA supplement groups showed similar cell survival rates, which were comparable to that in the control group (Figure 5A). Notably, BSA addition significantly increased the percentage and number of CD41a^+^ and CD41a^+^CD42b^+^ cells and the total number of suspended single cells derived from EBs compared to that in the non‐BSA control group (Figure 5B,C). The PVA addition in stage 3 led to a cellular yield of (12.2 ± 2.95) × 10^6^ CD41a^+^ CD42b^+^ MKs/1 × 10^6^ hESCs, showed a similar effect to BSA on these criteria (Figure 5B,C), indicating that PVA can be used to replace BSA in support of MK differentiation. We then analysed the biological safety of the suspended single cells obtained from clinical‐grade hESCs after 3D induction towards MKs. A series of test results showed that the differentiated cells derived from hESCs after MK lineage induction were negative for bacteria, fungi, mycoplasma and serious pathogenic microorganisms such as HIV, HBV, HCV and several other viruses (Table 1). BSA was undetected, and no endotoxin and cytokine residues were detected in the supernatants of the suspended single cells after washing 3 times. We also evaluated the safety of the differentiated cells in vivo. The mice injected with these cells via the blood or subcutaneously showed no teratoma formation after 8‐week inoculation. The organs of these mice, such as bone marrow, liver, heart, brain, and spleen, showed normal morphology and tissue structure (data not shown). However, significant tumours were found in mice inoculated with HeLa cells (Figure S3). These results indicate that suspended single cells generated from hESCs after 3D induction are clinically biosafe.

**TABLE 1:** Biological safety analysis of the clinical hESC‐derived MKs

Sterility and Pathogen	Method	Q‐CTS‐MK
Bacteria	Cultivation	‐
Fungi	Cultivation	‐
Mycoplasma	Cultivation	‐
HIV	ELISA	‐
HBV	ELISA/qPCR	‐
HCV	ELISA/qPCR	‐
HCMV	ECLIA	‐
HTLV I/II	ELISA	‐
HSV	qPCR	‐
EBV	ELISA	‐
HPV	qPCR	‐
Parvovirus B19	ELISA	‐
TPHA	Agglutination reaction	‐
Endotoxin level	Agglutination reaction	<1 EU/mL
BSA level	ELISA	‐
Abnormal proliferation	Soft agar assay	‐
Tumorigenicity	Nude mice inoculation	‐

### 3.6 CellSTACK‐cultured MKs induced from hESCs produce proPLTs and PLTs

**FIGURE 6:**
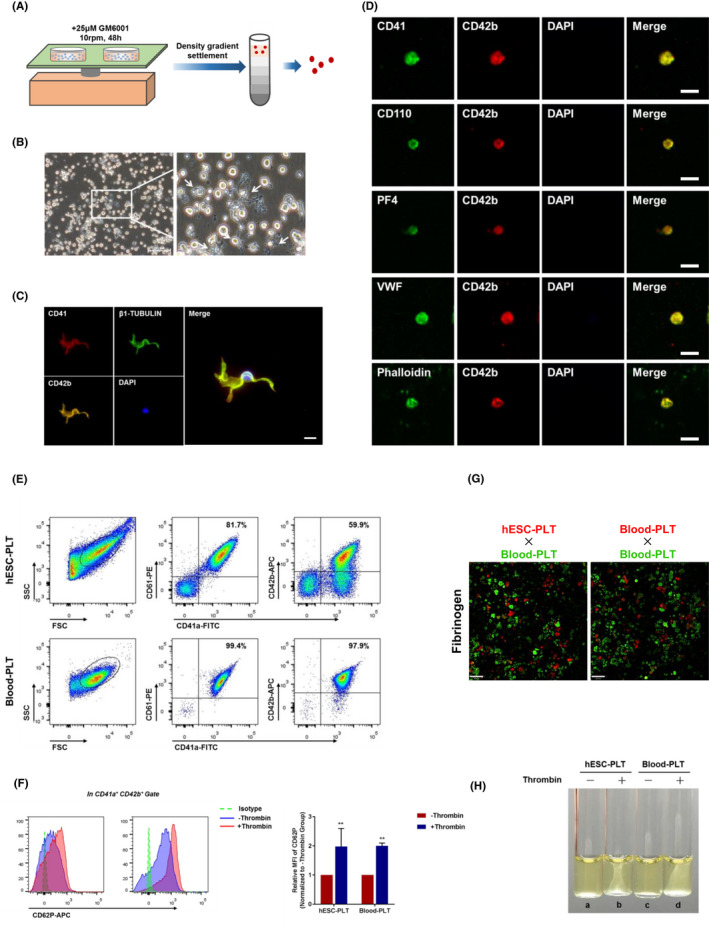
Functional characterization of platelets generated from hESCs in vitro. A, A schematic of platelet differentiation and isolation from hESC‐derived MK cells. B, Phase contrast images of proPLTs during platelet differentiation. Scale bars represent 100 μm. C, Immunostaining analysis for CD41, CD42b and β1‐tubulin in proPLTs during PLT differentiation process. Scale bars represent 10 μm. D, Immunofluorescence images of CD41, CD110, CD42b, PF4 and VWF proteins, and phalloidin staining in hESC‐derived PLTs. Scale bars represent 4 μm. E, Flow cytometry analysis of forward scatter (FSC) and side scatter (SSC), and CD41a, CD61 and CD42b expression on hESC‐derived PLTs and blood PLTs. F, Flow cytometry analysis of CD62P exposure on hESC‐derived PLTs and platelet concentrates in the presence or absence of thrombin stimulation. Relative MFI fold changes are shown normalized to the (‐) thrombin group. *P < .05, **P < .01 compared with the (‐) thrombin group. G, Aggregation response of hESC‐derived PLT and platelet concentrates in fibrinogen‐coated microwells. Scale bars represent 70 μm. H, Clot formation and retraction analysis of hESC‐derived PLTs and blood platelets

To investigate whether these MKs derived from hESCs in a 3D induction manner can differentiate into proplatelets (proPLTs) and PLTs, the cells were transferred into maturation medium with SCF, TPO, and GM6001 and rotated on a plate at 10 rpm for 48 hours (Figure 6A). We found that some MKs began to develop into proPLT‐forming cells, exhibiting thick cytoplasmic projections (Figure 6B). The proPLT‐forming cells expressed CD41, CD42 and β1‐TUBULIN (Figure 6C), indicating the existence of a proPLT‐forming cell phenotype. The supernatants were collected for PLTs, and their PLT characteristics were evaluated by immunofluorescence staining. The results showed that the induced PLTs strongly expressed CD42 and were co‐expressed with CD41, CD110, PF4, VWF and phalloidin (Figure 6D). We used flow cytometry to analyse the surface markers of these differentiated PLTs. We found that >80% of the cell debris was co‐expressed with CD41a and CD61, and >50% of the cell debris co‐expressed with CD41a and CD42b (Figure 6E), which showed a phenotype similar to that of peripheral blood PLTs. These PLTs presented similar aggregation capacity on fibrinogen compared with blood PLTs (Figure 6G). After thrombin stimulation, both the induced and natural PLTs showed increased CD62P expression and clot aggregation (Figure 6F,H), indicating that an active state of PLTs could be induced by thrombin.

## 4 DISCUSSION

The frequency of MK cells in bone marrow is only about 0.01 % of total nucleated cells, and they are derived from hematopoietic stem and progenitor cells.^27^ MK is the seed cell that generates PLTs and maintains normal platelet levels in peripheral blood by generating up to 3000 PLTs per MK.^28^ In certain disease conditions, such as thrombocytopenia caused by various factors, transfusion of in vitro stem cell‐differentiated MKs or PLTs has been developed into an acceptable supporting therapy approach to avoid bleeding.^29^, ^30^ For the clinical application of induced MK or PLT transfusion, it is critical to develop a practical protocol for the large‐scale generation of MKs and PLTs from stem cells in vitro.

Pluripotent stem cells, such as hESCs or hiPSCs, are the most promising cell resource for in vitro MK or PLT generation owing to their scalability and three‐germ layer differentiation capacity compared to other kinds of stem cells. To comply with the GMP standards, we adopted a GMP‐grade ES cell line, Q‐CTS‐hESC‐2, as the seed cells to produce MKs. Previously, Zhou laboratory had established two clinical‐grade hESC lines (Q‐CTS‐hESC‐1 and Q‐CTS‐hESC‐2) under cGMP conditions.^19^ Q‐CTS‐hESC‐2 cells have been used to prepare several differentiated clinical‐grade cell types, such as neurons, retinal pigment epithelium, cardiomyocytes and hepatocytes.^31^, ^32^, ^33^, ^34^ Here, we first established a feeder‐free, stepwise and adherent induction protocol for MK cell generation from hESCs. By mimicking embryonic megakaryopoiesis, we induced hESCs to undergo the typical mesoderm progenitor cell, hemogenic cell and hematopoietic cell development process by using an optimal 3‐stage induction medium containing different cytokines with or without small molecules. We also applied an immunoselection technique to harvest CD34^+^ cells, which acted as the seed cells for hematopoietic and MK differentiation process at the end of stage 2 induction. Many researchers have used this method to improve the differentiation efficiency of hematopoietic cells from pluripotent stem cells.^35^, ^36^, ^37^ Both Q‐CTS‐hESC‐2 and H9 hESCs showed high differentiation efficiency of CD41a^+^ MKs. This adherent induction method is practical and avoids feeder cell corporations. However, the MK yield calculated from each hESC is very low, which might be related to the inserted immunobead selection step; it could also be due to the low cell culture density in the adherent culture manner.

To obtain high MK differentiation efficiency and yield, we chose a 3D suspension culture method to induce hESCs to differentiate into MKs. In recent years, 3D suspension cultures of hESCs have been proven as a feasible strategy for expanding hESCs.^38^, ^39^, ^40^ Spin EB induction was used to generate hematopoietic cells from ESCs by several laboratories.^20^, ^41^, ^42^ However, for the large‐scale production of hematopoietic cells, the manipulation of the spin EB method is relatively time‐consuming, inefficient and difficult to generate over 10^7^ MKs. We improved and enlarged the suspension induction method by using a polystyrene CellSTACK culture chamber with an ultra‐low attachment surface. Single hESC aggregated and formed EBs in the culture medium. By changing the induction medium in an orderly manner, these EBs gradually produced many round and small hematopoietic cells that spread around them. Suspension cultures and differentiation helped simplify the manipulation program for the generation of MKs from hESCs. Notably, the 3D suspension induction protocol could generate (3.4 ± 2.5) × 10^8^ CD41a^+^ MKs per CellSTACK culture chamber by using 1L differentiation medium 3 within 16‐18 days of cell culturing. It is feasible to use this type of culture system to generate a large quantity of MKs, and this process could be simplified by using automatic culture machines. In the future, automatic and standard manipulation of the MK differentiation process is important for industrial production of MKs and PLTs from pluripotent stem cells. The induced cells derived from hESCs in our culture system were shown to have the characteristics of MKs by using several evaluation criteria, including cell morphology, the expression of key MK genes, polyploid detection, biomarker expression, as well as, proPLT formation and PLT release. The hESC‐derived MKs in the 3D induction system displayed relatively mature characteristics compared to the differentiated cells in the adherent induction method. MKs derived from 3D induction presented stronger MK‐characteristic gene expression, including *NFE2*, *FOG1* and *β1‐TUBULIN* (Figure S4A). More cells expressing β1‐TUBULIN were detected in the 3D induction group than the monolayer induction group (Figure S4B), indicating that more mature MKs were produced from 3D induction system. These differentiated MK‐lineage cells from 3D induction system demonstrated lower CFU‐MK numbers and secreted more MK and PLT‐characteristic cytokines, such as thrombospondin‐1 and MMP9. Several studies indicated that inhomogeneous size of EBs or colonies limited the differentiation efficiency of target cells.^43^, ^44^ Thus, our 3D induction protocol has more improvement space to improve the homogeneity of EB and increase the yield of MKs, including introduction of biomechanical forces. PLTs generated from these induced MKs could be activated by specific agonist thrombin or fibrinogen, further suggesting the functionality of induced MKs and PLTs derived from hESCs. However, due to the low PLT yield and lack of suitable animal models to evaluate the function of human stem cell‐derived MKs, it is still difficult to observe the PLTs generated from human pluripotent stem cell‐derived MKs in vivo. An unmet challenge is to improve the PLT yield and function of pluripotent stem cell‐derived MKs by introducing new technique.^45^, ^46^ Besides, new animal model needs to be established for better evaluation of the functionality of human stem cell‐derived MKs and PLTs.

For the clinical transfusion of induced MKs or PLTs, the induction medium, manufacturing process, and quality of induced MKs from stem cells must be strictly controlled. To comply with GMP standards, we chose GMP‐grade hESCs as seed cells. Our 3D induction method can help generate MKs from GMP‐grade hESCs under serum‐free and feeder‐free conditions. To avoid xenogeneic contamination, BSA was replaced with PVA in the induction medium, which did not affect the differentiation efficiency of MKs from hESCs. For the clinical application of induced MKs, all cytokines used (research‐grade) during the culture process could be replaced with GMP‐grade cytokine products. The microorganisms and the cytokines supplemented in the medium were undetectable in the supernatants of induced MKs. These induced cells demonstrated no risk of tumour formation in nude mice, indicating the in vivo safety of induced MK transfusion. For clinical applications, induced MKs should be cryopreserved as seed cells to produce PLTs or can be transfused directly into patients who are at risk of bleeding. The induced MKs obtained using our protocol showed a good cell survival rate after cryopreservation.

In summary, we developed a defined 3D differentiation protocol for the generation of MKs from hESCs using a special polystyrene CellSTACK culture chamber. The 3D manufacturing system could efficiently generate large numbers of MKs from hESCs after three stages of orderly induction within a culture period of 16‐18 days. The induced cells derived from hESCs in our culture system were shown to have the characteristics of MKs, as well as, proPLT formation and PLT release. Importantly, we generated clinically applicable MKs from clinical‐grade hESC lines and analysed the biosafety of these cells. The current protocol is a simple, practicable and upscalable method that does not require feeder cells or genetic modification, further enhancing the clinical application of MKs or PLTs derived from pluripotent stem cells.

## CONFLICT OF INTEREST

The authors declare that they have no competing interests.

## AUTHOR CONTRIBUTIONS

The contributions of each author made to the study are specified as follows: YHL and XTP designed the study; BWZ, XMW, GCZ, LJH, SHW, LC, ZF and LW performed the experiments and collected the data; BWZ, XMW, YHL, JH, LW, XN, JFX and WY analysed the data; YHL and BWZ prepared the manuscript.

## Supporting information

Fig S1

Supplementary Material

Fig S2

Fig S3

Fig S4

## Data Availability

All data generated or analysed during this study are included in the published article and its supplementary information files.
