# Supplementary figures and images for: Large‐scale generation of megakaryocytes from human embryonic stem cells using transgene‐free and stepwise defined suspension culture conditions
Source: Cell Prolif. 2021 Feb 21;54(4):e13002. doi: 10.1111/cpr.13002 (PMC8016648; doi:10.1111/cpr.13002)

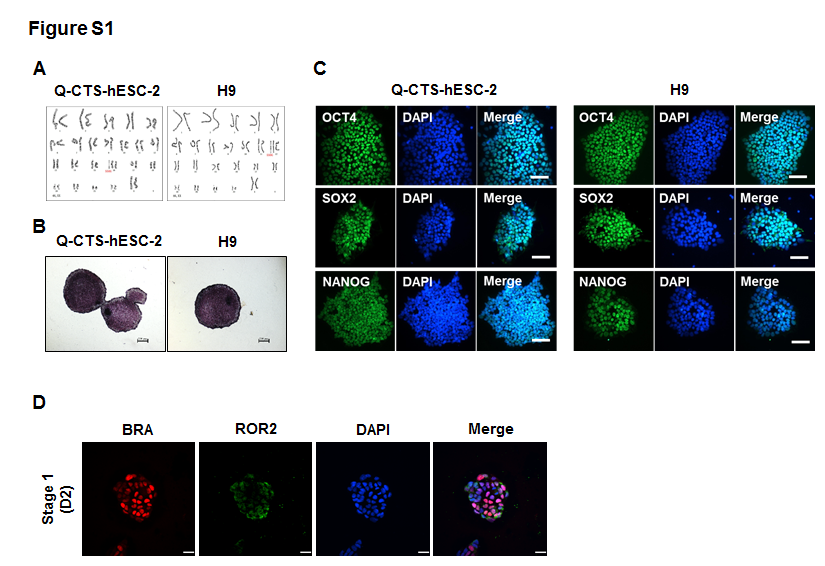

Supplement: Supplementary file 1 — Fig S1 [file CPR-54-e13002-s004.tif]

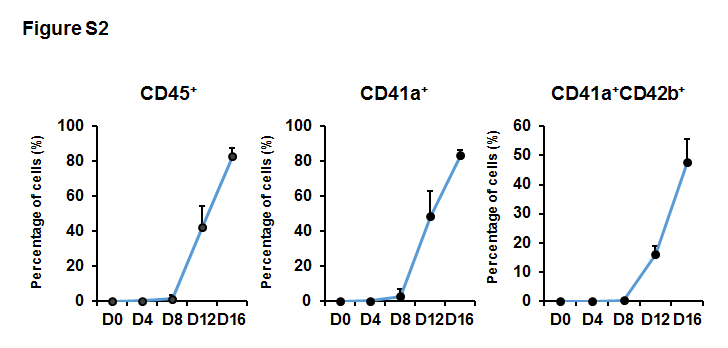

Supplement: Supplementary file 3 — Fig S2 [file CPR-54-e13002-s005.tif]

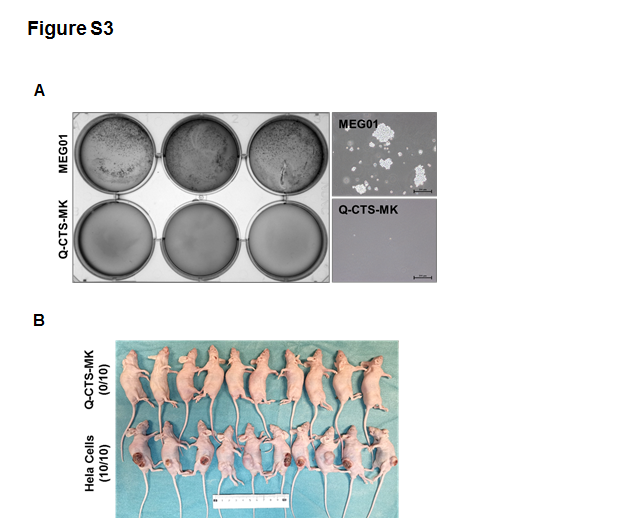

Supplement: Supplementary file 4 — Fig S3 [file CPR-54-e13002-s001.tif]

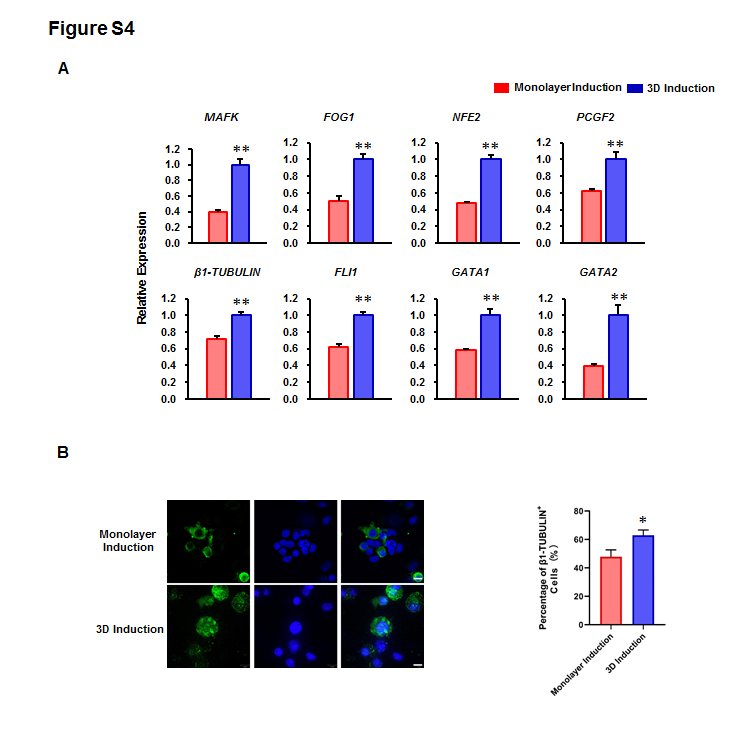

Supplement: Supplementary file 5 — Fig S4 [file CPR-54-e13002-s002.tif]
