# Supplementary material for: Large‐scale generation of megakaryocytes from human embryonic stem cells using transgene‐free and stepwise defined suspension culture conditions
Source: Cell Prolif. 2021 Feb 21;54(4):e13002. doi: 10.1111/cpr.13002 (PMC8016648; doi:10.1111/cpr.13002)
Supplement: Supplementary file 2 — Supplementary Material [file CPR-54-e13002-s003.docx]

**Supplemental Methods**

**Immunofluorescence Analysis**

The differentiated MKs were spun onto glass slides, fixed with 4% PFA, and permeabilized with 0.2% Triton-X100. After blocking with 10% donkey serum, the cells were stained with primary antibodies for overnight at 4 °C, followed by staining with Alexa Fluor 488/568/647-conjugated secondary antibody. The nuclei were stained with DAPI. Images were captured using a laser confocal microscope (Carl Zeiss). The antibody information is described in detail in Table S2.

**Gene Expression Analysis**

Total RNA from cultured cells was extracted using the RNeasy^TM^ Mini Kit (Qiagen) according to the manufacturer’s protocol. One microgram of RNA was used for cDNA synthesis using the ReverTra Ace^TM^ qPCR RT Master Mix (TOYOBO). qPCR analysis was performed using gene-specific primers with the SYBR^TM^ qPCR Mix (TOYOBO) on a CFX Connect^TM^ Real-time System (BIO-RAD). Gene expression levels were calculated using the minimal cycle threshold values (Ct) normalized to the expression of the internal control *GAPDH*. The primer sequences are described in detail in Table S3.

**Cytokine Array Analysis**

The differentiated MKs were cultured in BEL medium (1 × 10^6^/mL) for 24 h. Culture supernatants were collected and applied to the nitrocellulose membranes provided by Proteome Profiler Human XL Cytokine Array Kit (R&D systems, ARY022). The analysis was performed according to the manufacturer’s protocol. Image acquisition was performed using Amersham Imager 680 (GE Healthcare). Gray value analysis was performed using the Quantity One software.

**ELISA Analysis**

The differentiated MKs were cultured in BEL medium (1×10^6^/mL) for 24 hours. The culture supernatants were collected, and the dead cells were eliminated by centrifugation. The concentrations of secreted cytokines were determined by ELISA kits according to the manufacturer’s protocol. The ELISA kit information is described in detail in Table S2.

**Karyotype Analysis**

Q-CTS-hESC-2 and H9 cells were used for karyotype analysis after 3 months of culture. Karyotype and G-binding were analyzed at KingMed Diagnostics (Beijing, China).

**Biosafety Evaluation**

Information of tested items is listed in Table 1. The samples were analyzed at KingMed Diagnostics (Beijing, China). The ‘‘Pharmacopoeia of the People’s Republic of China’’ was used as a reference for the testing methods.

**Supplemental Tables**

**Table S1 The composition of BEL medium**

| **Media Component** | **Component Content** |
| --- | --- |
| IMDM/F-12 | 1:1 |
| Deionized BSA | 2.5 mg/ml |
| Synthechol Solution | 0.2 % |
| Linoleic Acid | 100 ng/ml |
| Linolenic Acid | 100 ng/ml |
| Ascorbic Acid 2-phosphate | 50 μg/ml |
| α-MTG | 350–450 μM |
| Glutamax I | 2 mM |
| Protein-Free Hybridoma Mix | 5 % |
| Insulin-Transferrin-Selenium | 1 % |

**Table S2. Key Resources Table**

| **Antibodies** | **Source** | **Identifier** |
| --- | --- | --- |
| Anti-hCD34-PECy7 | BD | Cat#560710 |
| Anti-hCD45-PE | BD | Cat#555483 |
| Anti-hCD45-APC | BD | Cat#555485 |
| Anti-hCD41a-APC | BD | Cat#559777 |
| Anti-hCD41-BV421 | Biolegend | Cat#303730 |
| Anti-hCD61-FITC | eBioscience | Cat#11-0619-42 |
| Anti-hCD42b-PE | eBioscience | Cat#12-0429-42 |
| Anti-hCD62P-APC | eBioscience | Cat#12-0628-71 |
| Rabit anti-CD41 | Abclonal | Cat#A11490 |
| Rabit anti-VWF | Millipore | Cat#AB7356 |
| Mouse anti-β1-TUBULIN | R&D | Cat#MAB8527 |
| Rabit anti-OCT4 | Abcam | Cat# ab271937 |
| Rabit anti-SOX2 | Abcam | Cat#ab97959 |
| Rabit anti-NANOG | Abcam | Cat#ab109250 |
| Goat anti-BRA | R&D | Cat#AF2085 |
| Rabit anti-ROR2 | Abcam | Cat#2722-1 |
| Rabit anti-CD110 | Abcam | Cat#ab232755 |
| Mouse anti-CD42b | BD | Cat#555471 |
| Rabit anti-PF4 | BOSTER | Cat#BA4122 |
|  |  |  |
| **Chemicals and Recombinant Proteins** | **Source** | **Identifier** |
| SCF | Peprotech | Cat#300-07 |
| Flt-3L | Peprotech | Cat#300-19 |
| IL-3 | Peprotech | Cat#200-03 |
| TPO | Peprotech | Cat#300-18 |
| VEGF | R&D | Cat#293-VE |
| IGF-1 | R&D | Cat#291-G1 |
| bFGF | Peprotech | Cat#100-18c |
| IL-11 | Peprotech | Cat#200-11 |
| BMP-4 | R&D | Cat#314-BP |
| Activin A | R&D | Cat#338-AC |
| VTN-N | Gibco | Cat#A14700 |
| SB431542 | Selleck | Cat#S1067 |
| CHIR99021 | Selleck | Cat#S2924 |
| Y27632 | Selleck | Cat#S1049 |
|  |  |  |
| **ELISA Kit** | **Source** | **Identifier** |
| PF4 | BOSTER | Cat#EK0726 |
| VWF | BOSTER | Cat#EK1743 |
| IL-8 | Solarbio | Cat#SEKH-0016 |
| THBS-1 | Solarbio | Cat#SEKH-0322 |
| MMP9 | NEOBIOSCIENCE | Cat#EHC115 |
|  |  |  |
| **Culture Medium** | **Source** | **Identifier** |
| mTeSR | Stemcell | Cat#85850 |
| TeSR-E8 | Stemcell | Cat#05990 |

**Table S3. Primer**

| **Gene Name** | **Forward** | **Reverse** |
| --- | --- | --- |
| *OCT4* | GTGTTCAGCCAAAAGACCATCT | GGCCTGCATGAGGGTTTCT |
| *SOX2* | GCCGAGTGGAAACTTTTGTCG | GGCAGCGTGTACTTATCCTTCT |
| *GATA2* | GCAACCCCTACTATGCCAACC | CAGTGGCGTCTTGGAGAAG |
| *RUNX1* | CTGCCCATCGCTTTCAAGGT | GCCGAGTAGTTTTCATCATTGCC |
| *FLI1* | CAGCCCCACAAGATCAACCC | CACCGGAGACTCCCTGGAT |
| *GATA1* | CTGTCCCCAATAGTGCTTATGG | GAATAGGCTGCTGAATTGAGGG |
| *FOG1* | CGTGCTTCGAGTGCGAGAT | GGCCTGAACAGTAGAGGCG |
| *NFE2* | GCAGGAACAGGGTGATACAGC | GCAGCTCGGTGATGGACAT |
| *β1-TUBULIN* | AACACGGGATCGACTTGGC | CTCGGGGCACATATTTCCTAC |
| *PF4* | AGCCTGGAGGTGATCAAGG | CCATTCTTCAGCGTGGCTA |
| *TIE1* | AAGCAGACAGACGTGATCTGG | GCACGATGAGCCGAAAGAAG |
| *ERG* | CGTGCCAGCAGATCCTACG | GGTGAGCCTCTGGAAGTCG |
| *MAFK* | *GAGAGTGGCTCACACGTCG* | *CTGCTCACCGTCAAATGATGG* |
| *CXCL8* | TTTTGCCAAGGAGTGCTAAAGA | AACCCTCTGCACCCAGTTTTC |
| *SPP1* | CTCCATTGACTCGAACGACTC | CAGGTCTGCGAAACTTCTTAGAT |
| *CHI3L1* | GTGAAGGCGTCTCAAACAGG | GAAGCGGTCAAGGGCATCT |
| *CCL2* | CAGCCAGATGCAATCAATGCC | TGGAATCCTGAACCCACTTCT |
| *SERPINE1* | ACCGCAACGTGGTTTTCTCA | TTGAATCCCATAGCTGCTTGAAT |
| *MMP9* | TGTACCGCTATGGTTACACTCG | GGCAGGGACAGTTGCTTCT |
| *PLAUR* | TGTAAGACCAACGGGGATTGC | AGCCAGTCCGATAGCTCAGG |
| *GAPDH* | GAGTCAACGGATTTGGTCGT | TTGATTTTGGAGGGATCTCG |

**Supplemental Figure Legends**

**Figure S1.** Characterization of human embryonic stem cells (hESCs) in a feeder-free culture. A, Karyotype analysis of hESCs after three months of culture. B, Alkaline phosphatase (AP) activity analysis of hESCs. C, Immunostaining of hESC with pluripotency markers OCT4, SOX2, and NANOG; the nuclei were stained with DAPI. D. Mesoderm-specific proteins expression was detected during hESC mesoderm differentiation (Stage 1) by immunostaining. The nuclei were counterstained with DAPI.

**Figure S2.** Percentages of CD45^+^, CD41a^+^, and CD41a^+^CD42b^+^ cells in suspended single cell population during the differentiation process.

**Figure S3.** Biological safety analysis of clinical hESC-derived MKs. A, Soft agar assay for colony formation. MEG01 cells were used as the positive control. B, Subcutaneous tumorigenicity test. HeLa cells were used as the positive control.

**Figure S4.** Characterization of MKs generated from monolayer induction and 3D induction. A, Quantitative RT-PCR analysis of megakaryocytic marker genes expressed in suspension cells from monolayer induction and 3D induction. B, the percentage of β1-TUBULIN+ cells in suspended single cell population generated from the monolayer induction or 3D differentiation model by immunofluorescence staining. Scale bars represent 10 μm.
